# Combinations of job demands are associated with increased risk of depression in clinical veterinary practice: a cross-sectional study

**DOI:** 10.1186/s13620-024-00284-x

**Published:** 2024-12-27

**Authors:** Simone Vestergaard Christiansen, Thomas Clausen

**Affiliations:** National Research Centre for the Working Environment, Lersoe Parkalle 105, Copenhagen, DK-2100 Denmark

**Keywords:** Mental health, Psychosocial work environment, Stress, Emotional demands, Compassion fatigue

## Abstract

**Background:**

Veterinarians have a high prevalence of mental health disorders, such as depression. Previous research suggests that veterinarians are highly exposed to emotional demands at work and that these emotional demands are associated with adverse mental health outcomes. However, little is known about the consequences of the simultaneous exposure to emotional demands and other types of job demands in clinical veterinary practice. In this cross-sectional study, we investigate the combined effect of simultaneous exposure to emotional demands and other types of job demands on the risk of depression. We invited 1,757 employees in clinical veterinary practice in Denmark to participate in an online survey in the spring of 2022.

**Results:**

We obtained response from 885 employees (50.4%). Mean age was 38.2 years and 90.2% of the sample identified as women. The majority of the respondents worked in small animal practice (80.6%). We assessed psychosocial job demands (emotional demands, quantitative demands, role conflicts, work pace, and threats) and depressive symptoms in the study questionnaire, and defined depression as a score of ≥ 21 on the Major Depression Inventory. Data were analyzed using logistic regression analysis. 15.1% of the participants had an indication of depression. Results showed an increased risk of depression for participants reporting high emotional demands in combination with high quantitative demands (OR:8.37; 95%CI:4.31–16.24), high role conflicts (OR:8.95; 95%CI:4.71–16.99), threats at work (OR:7.06; 95%CI:4.06–12.28) and high work pace (OR:14.24; 95%CI:6.51–31.15). The combined effects indicated additive but not synergistic interaction.

**Conclusions:**

Combinations of emotional demands and other types of job demands are associated with an increased risk of depression among employees in clinical veterinary practice in Denmark. The results have implications for preventing negative health-related consequences of adverse psychosocial working conditions among employees in clinical veterinary practice. Preventive strategies and initiatives to promote a healthy psychosocial work environment and well-being among veterinary employees are discussed, and we further encourage employers and relevant authorities in veterinary practice to prioritize efforts to enhance the psychosocial work environment and employee well-being in clinical veterinary practice.

**Supplementary Information:**

The online version contains supplementary material available at 10.1186/s13620-024-00284-x.

## Introduction

Mental health disorders such as depression and anxiety are highly prevalent in the working population worldwide [1, 2]. Previous studies have shown associations between psychosocial working conditions and adverse health-related outcomes such as depression, sickness absence, and stress-related disorders [3–7]. More specifically, emotional demands at work have been found to predict adverse mental health outcomes, including depression [4, 8, 9]. These findings are also prevalent in the veterinary profession, as extant research suggests that employees in clinical veterinary practice are highly exposed to emotional demands at work [10, 11] and further that these emotional demands are associated with adverse outcomes, such as lower job satisfaction and mental health disorders among veterinarians, veterinary nurses, and animal care employees in general [10, 12–17]. These emotional demands may for example be due to experiences from encounters with pet owners that are emotionally difficult to handle [11] and moral and ethical dilemmas, e.g. when performing euthanasia [10].

Due to the potential emotional load in interactions between veterinary staff and pet owners [10–12], emotional demands may be considered a central job demand for employees in clinical veterinary practice. Following the Job Demands-Resources Model (JD-R), job demands are those aspects of the job that require sustained physical or psychological effort, and which consequently may be associated with psychological and/or physical strain [18]. The JD-R model also proposes that high levels of job demands may exhaust employees both physically and psychologically, which again may lead to adverse health-related outcomes [18, 19].

Following the Conservation of Resources (COR) theory [20], employees invest their resources to deal with exigent job demands, and high levels of one job demand may consequently reduce the capacity of employees to deal successfully with high levels of additional job demands [21]. This may again lead to an increased risk of adverse health-related outcomes such as depression. Previous studies do indeed suggest that the simultaneous exposure to high levels of multiple job demands may be associated with an increased risk of adverse work- or health-related outcomes when compared to a work environment in which workers report exposure to high levels of only one or no job demands [22–26].

Against this background, we hypothesize that simultaneous exposure to multiple job demands is associated with an increased risk of depression for employees in clinical veterinary practice. Since emotional demands are prevalent in clinical veterinary practice [10–12], the aim of this study is, therefore, to explore how the following combinations between emotional demands and other types of job demands are associated with the risk of depression among employees in clinical veterinary practice:

- Emotional demands and quantitative demands.
- Emotional demands and role conflicts.
- Emotional demands and threats at work.
- Emotional demands and work pace.

We have included quantitative demands, role conflicts, threats at work, and work pace in the analyses as these exposures are prevalent in clinical veterinary practice [27]. Moreover, previous studies have found that exposure to these job demands was associated with an increased risk of depression [28–30].

By focusing on the association between combinations of job demands and depression in clinical veterinary practice, this study adds to the literature on two counts. First, the study adds to the literature on associations between combinations of job demands and health-related outcomes, such as depression. Second, the study contributes to our knowledge on the consequences of adverse psychosocial working conditions in veterinary practice. This knowledge may support the veterinary profession in preventing adverse mental health outcomes, such as depression.

## Materials and methods

### Study design

The present study is based on a cross-sectional study design. The survey was administered by email and we obtained email addresses of potential participants with assistance from The Danish Veterinary Association (DVA) and the veterinary clinics themselves. The DVA sent a newsletter to clinics that were members of the DVA and encouraged them to submit a list of their employees including clinic owners, employed veterinarians, veterinary nurses, veterinary nurse students, and other staff. In Denmark, veterinarians are organized within the DVA. The DVA has approximately 4,400 members of which roughly 70% are women [31]. Veterinary nurses and veterinary nurse students are organized within the Veterinary Nurses Union (VNU). The VNU has about 1100 members, of which 99% are women [32].

In the spring of 2022, we sent invitations to participate in the survey by email to 1,757 employees in clinical veterinary practice and we collected data via an online survey. After two weeks, we sent reminders to employees, who had not responded to the questionnaire. In the invitation letter to the survey, we carefully described the purpose of the data collection and clearly stated that participation was voluntary. Since participation in the survey was voluntary, we interpret the decision of individual participants to fill in the questionnaire as informed consent. In addition, the survey was anonymous and it was possible for participants to leave items blank. Participants received no rewards for participating in the survey. The data collection was approved by the Danish Data Protection Agency. In Denmark, approval from Ethics Committees is not required for survey research. In all, 885 participants participated in the survey (response rate: 50.4%), which implies that approximately 16% of the estimated background population of 5,500 veterinarians and veterinary nurses are included in the survey.

The study questionnaire included 109 questions divided into the following four sections; first, a section collecting occupational background information such as job position, type of practice, seniority, and working hours; second, a section assessing psychosocial work environment and workers’ reactions to the work situation using the Danish Psychosocial Work Environment Questionnaire (DPQ) [33]; third, a section assessing depressive symptoms using the Major Depression Inventory (MDI) [34] and other health-related questions about sickness-absence and consumption of alcohol and medication; finally, a section collecting demographic background information about gender identity, age, civil status and cohabitation with children. Of these four areas, the present study includes data on occupational and demographic background characteristics, five types of job demands within the psychosocial work environment, and symptoms of depression. The independent and dependent variables of interest will be described below. Results from the survey have previously been published in a comprehensive Danish report [27].

### Independent variables: psychosocial job demands

We collected data on psychosocial working conditions using the Danish Psychosocial Work Environment Questionnaire (DPQ) [33]. The psychometrics properties of the DPQ have been described elsewhere [28, 33]. We used four multi-item scales from the DPQ in the present study (all items are reported in Appendix 1).

*Emotional demands* were measured by three items (Cronbach’s alpha: 0.81). An example item is: “*Are you placed in emotionally demanding situations at work*?”

*Quantitative demands* were measured by four items (Cronbach’s alpha: 0.87). An example item is: “*How often is it the case that you do not have time to complete all your work tasks*?”

*Role conflicts* were measured by four items (Cronbach’s alpha: 0.79). An example item is: “*Are there any conflicting demands in your work*?”

*Work pace* was measured by two items (Pearson’s *r*: 0.60). An example item is: “*Do you have to work very fast*?”

Response options for items about emotional demands, quantitative demands, and work pace were: (5) Always (4) , Often (3) , Sometimes (2) , Rarely, and (1) Never/almost never, while response options for items about role conflicts were: (5)  To a very large extent (4) , To a large extent (3) , Somewhat (2), To a small extent, and (1) To a very small extent). For each multi-item scale, we added items into scales and computed average scale scores ranging from 1 to 5. For analytical purposes, we coded the four scales as categorical variables, by coding the upper median on these scales as being *exposed* to high emotional demands, high quantitative demands, high role conflicts, and high work pace.

We measured threats at work with the following item from the DPQ: “*Have you been exposed to work-related threats during the last 12 months?*”. In the questionnaire, we provided the following definition of threats to the participants: ’Threats’ denotes verbal or written threats or threatening behavior. For analytical purposes, response options were dichotomized into (1) exposed (‘Daily’, ‘Weekly’, ‘Monthly’, and ‘Now and then’) and (2) not exposed (‘Never’).

### Dependent variable: major depression inventory

We collected data on depression using the validated questionnaire *Major Depression Inventory* (MDI) [34]. The questionnaire consisted of 12 items (10 symptoms) that measure the participants’ mood ranging from (0) None of the time to (5) All of the time. Item examples are: “*How often over the past two weeks have you had less self-confidence*?”, and “*How often over the past two weeks have you felt that life was not worth living*?” (all items are reported in Appendix 1). The MDI-score is calculated from 10 of the 12 items and the sum score goes from 0 (no depression) to 50 (maximum depression). The clinically validated cut-point for a depressive disorder is an MDI-score ≥ 21 and, therefore, we coded respondents with an MDI-score ≥ 21 as cases of depression [34].

### Covariates

All analyses were adjusted for gender, age, job group, and type of practice. Information on these variables were collected in the study questionnaire.

### Statistical analysis

Associations between the independent variables (job demands) and the dependent variable (depressive disorder) was analyzed using the LOGISTIC procedure in SAS 9.4 (SAS Institute, Cary, NC). Using logistic regression analyses, we calculated Odds Ratios (OR) and 95% Confidence Intervals (95% CI) for the association between different combinations of job demands and risk for depression. Estimates were adjusted for covariates mentioned above. We also used Pearson’s correlation coefficient to assess the pairwise associations between the independent variables.

We followed the approach described by Andersson et al. [35] and analyzed the interaction between two independent categorical variables by investigating deviation from additivity from the sum of the risk estimates of the two independent variables. We estimated ORs for four combinations of exposure levels of two different job demands. An example: employees are exposed to high or low levels of two job demands (job demand A and job demand B), which creates four exposure groups: (1) Both job demand A and B at low level (reference, OR_00_ = 1), (2) job demand A at high level and job demand B at low level (OR_10_), (3) job demand A at low level and job demand B at high level (OR_01_) and (4) both job demand A at high level and job demand B at high level (OR_11_). We tested if the estimate of OR_11_ deviated significantly from additivity by calculating the relative excess risk due to interaction (RERI). RERI-values that are significantly different from 0 indicate deviation from additivity [35]. Deviation from additivity means that exposure to high levels of two job demands are associated with an increased health risk that goes beyond the additive effect of the risk associated with each of the two job demands. This implies that the excess risk is due to an interaction effect between the two job demands. For example, this means that the combined risk of high emotional demands and high work pace for depression is significantly higher than the sum of the risk estimates that is respectively ascribed to high emotional demands and high work pace [36]. This is also called synergistic effects. In the logistic regression analyses, we also computed the Somers’ D-coefficient to assess the model fit between independent and dependent variables. The Somers’ D is suitable for categorical variables and ranges from − 1 to 1. Values close to 1 or -1 indicate a strong association, while values closer to 0 indicate weaker associations between the variables. In all analyses, the significance level is set to 5%.

## Results

### Descriptive statistics

Table 1 shows descriptive statistics for background variables and symptoms of depression. The mean age was 38.2 years and 90.2% of the study population identified as women. Further, 42.8% of the respondents were employed veterinarians, 29.7% were veterinary nurses and 80.6% of the respondents work in small animal practice. Finally, the results show that 15.1% (95% CI: 12.6 to 17.6) of the population have indications of depressive symptoms.

**Table 1:** Descriptive statistics for background variables

	*N*	%	Mean
Total	885	100	
**Gender**	**787**		
Male	75	9.5	
Female	712	90.2	
**Age**	**791**		**38.2**
< 25 years	45	5.7	
25–34 years	303	38.3	
35–44 years	220	27.8	
45–54 years	147	18.6	
55–64 years	70	8.9	
> 65 years	6	0.8	
**Job position**	**885**		
Clinical owner	85	9.6	
Employed veterinarian	379	42.8	
Veterinary nurse	263	29.7	
Veterinary nurse student	84	9.5	
Other	74	8.4	
**Type of practice**	**878**		
Equine practice	38	4.3	
Small animal practice	708	80.6	
Production practice	46	8.3	
Mixed practice	86	9.8	
**Depressive symptoms (MDI-score)**	**789**		
MDI ≥ 21	119	15.1	

*Note*: N may vary due to missing values

Table 2 shows that participants reporting exposure to high quantitative demands (OR 2.9; 95% CI 1.9 to 4.6), high emotional demands (OR 4.2; 95% CI 2.7 to 6.6), high role conflicts (OR 3.4; 95% CI 2.1 to 5.2), exposure to threats at work (OR 3.1; 95% CI 2.0 to 4.8), and high work pace (OR 3.8; 95% CI 2.4 to 6.1) have statistically significant increased risk of depression when compared to participants reporting low exposure to each of the five job demands. Appendix 2 presents the results on associations between study covariates (gender, age, job group, and type of practice) and risk of depression.

**Table 2:** Risk of depression for exposure to high job demands: results from logistic regression analysis

	At risk *N* (%)	Cases *N* (%)	OR	95% CI
**Quantitative demands**				
High exposure	390 (49.4)	87 (11.0)	2.9	1.9 to 4.6
Low exposure	399 (50.6)	32 (4.1)	1	Ref.
**Emotional demands**				
High exposure	334 (42.4)	85 (10.8)	4.2	2.7 to 6.6
Low exposure	453 (57.6)	34 (4.3)	1	Ref.
**Role conflicts**				
High exposure	360 (45.6)	87 (11.0)	3.3	2.1 to 5.2
Low exposure	429 (54.4)	32 (4.1)	1	Ref.
**Threats at work**				
High exposure	205 (26.0)	55 (7.0)	3.1	2.0 to 4.8
Low exposure	584 (74.0)	64 (8.1)	1	Ref.
**Work pace**				
High exposure	369 (46.8)	91 (11.5)	3.8	2.4 to 6.1
Low exposure	420 (53.2)	28 (3.6)	1	Ref.

*Note*: All analyses are adjusted for gender, age, job position, and type of practice

### Combination of emotional demands and quantitative demands

**Fig. 1:**
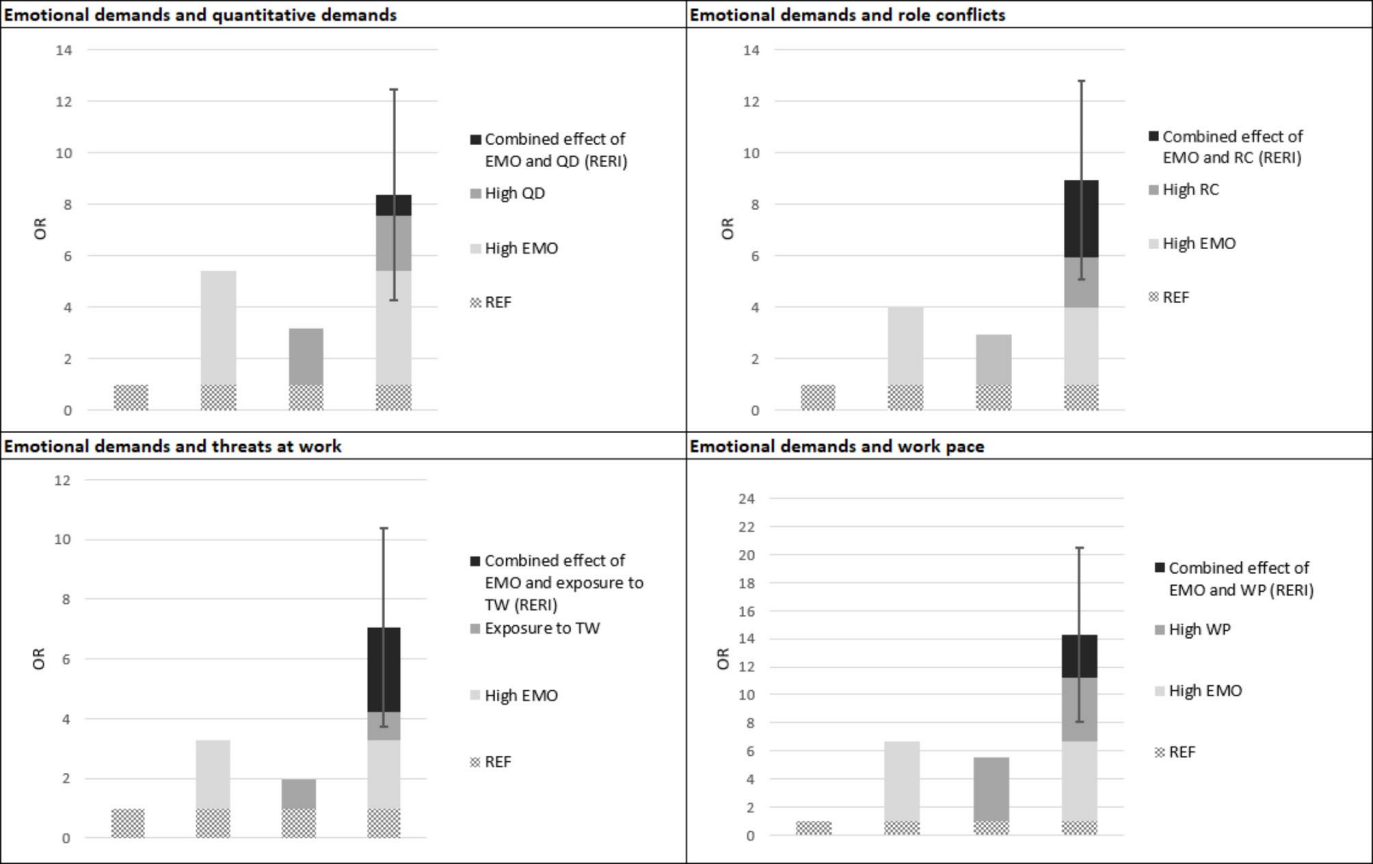
Plots of associations between four combinations of job demands and risk of depression: Results from logistic regression analyses. *Note*: EMO, emotional demands; QD, quantitative demands; RC, role conflicts; WP, work pace; TW, threats at work

Table 3 shows that respondents reporting exposure to high emotional demands and low quantitative demands (OR 5.41; 95% CI 2.50 to 11.71) and high quantitative demands and low emotional demands (OR 3.17; 95% CI 1.49 to 6.73) have increased risk of depression when compared to the unexposed reference group (see also Fig. 1). Respondents reporting exposure to a combination of high emotional demands *and* high quantitative demands have a higher risk of depression (OR 8.37; 95% CI 4.31 to 16.24) than respondents reporting exposure to only one of the two exposures. However, the combined effect of exposure to these two job demands does not depart from additivity as the RERI-coefficient is not statistically significant (RERI 0.80; 95% CI -3.30 to 4.89). A Somers’ D coefficient of 0.531 indicates a satisfactory model fit.

### Combination of emotional demands and role conflicts

Table 3 shows that respondents reporting exposure to high emotional demands and low role conflicts (OR 3.97; 95% CI 1.88 to 8.41) and high role conflicts and low emotional demands (OR 2.94; 95% CI 1.40 to 6.18) have increased risk of depression when compared to the unexposed reference group (see also Fig. 1). Respondents reporting exposure to a combination of high emotional demands *and* high role conflicts have a higher risk of depression (OR 8.95; 95% CI 4.71 to 16.99) than respondents only reporting exposure to one of the two exposures. The combined effect of exposure to the two job demands does not depart from additivity as the RERI-coefficient is not statistically significant (RERI 3.03; 95% CI -0.84 to 6.90). A Somers’ D coefficient of 0.551 indicates a satisfactory model fit.

### Combination of emotional demands and threats at work

Table 3 shows that respondents reporting exposure to high emotional demands and no exposure to threats at work (OR 3.27; 95% CI 1.86 to 5.74) and exposure to threats at work and low emotional demands (OR 1.95; 95% CI 0.79 to 4.84) have a higher risk of depression when compared to the unexposed reference group (see also Fig. 1). It must be noted, though, that the latter association is statistically non-significant. Respondents reporting exposure to a combination of high emotional demands *and* exposure to threats at work have a higher risk of depression (OR 7.06; 95% CI 4.06 to 12.28) than respondents reporting exposure to one of the two exposures. The combined effect of exposure to the two job demands does not depart from additivity as the RERI-coefficient is not statistically significant (RERI 2.84; 95% CI -0.50 to 6.17). A Somers’ D coefficient of 0.563 indicates a satisfactory model fit.

### Combination of emotional demands and work pace

Table 3 shows that respondents reporting exposure to high emotional demands and low work pace (OR 6.71; 95% CI 2.83 to 15.92) and high work pace and low emotional demands (OR 5.54; 95% CI 2.39 to 12.82) have a higher risk of depression when compared to the unexposed reference group (see also Fig. 1). Respondents reporting exposure to a combination of high emotional demands *and* high work pace have a higher risk of depression (OR 14.24; 95% CI 6.51 to 31.15) than respondents reporting exposure to one of the two exposures. The combined effect of exposure to the two job demands does not depart from additivity as the RERI-coefficient is not statistically significant (RERI 2.99; 95% CI -3.22 to 9.20). A Somers’ D coefficient of 0.526 indicates a satisfactory model fit.

**Table 3:** Risk of depression for combinations of job demands: results from logistic regression analysis

						At risk *N* (%)	Cases *N* (%)	OR	95% CI	RERI	95% CI	Somers’ D
**Combination: quantitative demands and emotional demands (Pearson’s** ***r*** **= 0.45)**										**0.80**	**-3.30 to 4.89**	**0.531**
Low quantitative demands and low emotional demands						294 (37.4)	12 (1.5)	1	Ref.			
High quantitative demands and low emotional demands						159 (20.2)	22 (2.8)	3.17	1.49 to 6.73			
Low quantitative demands and high emotional demands						103 (13.1)	20 (2.5)	5.41	2.50 to 11.71			
High quantitative demands and high emotional demands						231 (29.3)	65 (8.3)	8.37	4.31 to 16.24			
**Combination: role conflicts and emotional demands (Pearson’s** ***r*** **= 0.44)**										**3.03**	**-0.84 to 6.90**	**0.551**
Low role conflicts and low emotional demands						300 (38.1)	13 (1.7)	1	Ref.			
High role conflicts and low emotional demands						153 (19.4)	21 (2.7)	2.94	1.40 to 6.18			
Low role conflicts and high emotional demands						128 (16.2)	19 (2.4)	3.97	1.88 to 8.41			
High role conflicts and high emotional demands						206 (26.1)	66 (8.4)	8.95	4.71 to 16.99			
**Combination: threats at work and emotional demands (Pearson’s** ***r*** **= 0.45)**										**2.84**	**-0.50 to 6.17**	**0.526**
No exposure to threats and low emotional demands						397 (50.4)	26 (3.3)	1	Ref.			
Exposure to threats and low emotional demands						56 (7.1)	8 (1.0)	1.95	0.79 to 4.84			
No exposure to threats and high emotional demands						185 (23.5)	38 (4.8)	3.27	1.86 to 5.74			
Exposure to threats and high emotional demands						149 (18.9)	47 (6.0)	7.06	4.06 to 12.28			
**Combination: work pace and emotional demands (Pearson’s** ***r*** **= 0.40)**										**2.99**	**-3.22 to 9.20**	**0.563**
Low work pace and low emotional demands						291 (37.0)	8 (1.0)	1	Ref.			
High work pace and low emotional demands						162 (20.6)	26 (3.3)	5.54	2.39 to 12.82			
Low work pace and high emotional demands						127 (16.1)	20 (2.5)	6.71	2.83 to 15.92			
High work pace and high emotional demands						207 (26.3)	65 (8.26)	14.24	6.51 to 31.15			

*Note*: RERI was calculated using the following formula RERI = OR_11_-OR_10_-OR_01_ + 1. All analyses are adjusted for gender, age, job position, and type of practice

## Discussion

In this cross-sectional study of 885 employees in Danish clinical veterinary practice, we found that participants reporting exposure to combinations of high emotional demands and each of the other types of job demands had a higher risk of depression when compared to participants reporting exposure to one or none of the job demands that we investigated in this study. The results, therefore, suggest that the simultaneous presence of emotional demands in combination with other job demands are associated with an increased risk of depression among employees in clinical veterinary practice.

For all four combinations under study, we found that the increased risk of depression manifested itself through additive effects, which implies that we found no synergistic effects on depression in any of the four combinations of job demands. It must be noted, though, that we found comparatively high RERI-values for all four combinations, which indicates synergistic interaction between the investigated job demands. The results of the study imply that the association between emotional demands and the risk of depression is boosted when combined with high levels of other job demands and we identified two possible explanations for this. First, high levels of job demands (e.g. quantitative demands or role conflicts) may limit the ability of veterinary workers to handle emotionally laden encounters with pet owners, which again may increase the risk of conflicts with pet owners (e.g. threatening behaviors). These dynamics may contribute to increasing the level of emotional demands in specific work situations, which further increases the risk of depression. Second, high job demands (e.g. quantitative demands or work pace) may limit access to social support from colleagues and/or supervisors, which again implies that the possibilities to cope with emotionally demanding or even conflictual relations with pet owners are weakened when employees are simultaneously exposed to multiple job demands.

The findings of this study are in line with findings from previous studies suggesting that simultaneous exposure to multiple job demands increases the risk of adverse work- and health-related outcomes such as lower job satisfaction [26], increased risk of depression [24, 37], and long-term sickness absence [23, 24]. The findings of the present study are also in line with findings from other studies showing that veterinarians and employees in veterinary practice are at high risk of depression and decreased psychological well-being [13, 38] and that these ailments may be ascribed to conditions in the work environment such as emotional demands [12, 39]. These associations have also been found in other professions such as healthcare, education and social work [8, 40, 41].

According to the Conservation of Resources (COR) theory [20, 42], the results of this study indicate that employees in clinical veterinary practice facing high emotional demands, will need to invest psychological resources to cope with these emotional demands, which again may reduce their ability to deal successfully with high levels of additional job demands. The dynamics at play, when employees experience high levels of multiple job demands, are labelled ‘loss spirals’ and this concept may contribute to an explanation of the findings of this study. Namely that combinations of strain-inducing job demands may contribute towards a depletion of personal and work-related resources in individual employees thereby reducing the capacity of employees to deal successfully with future job demands. These dynamics may increase the risk of adverse health-related outcomes, such as depression, which again may intensify the perception of job demands in exposed employees, thereby leading to a downward spiral in the dynamic interplay between the psychosocial working conditions and the mental health of the exposed employees.

In the literature, numerous studies have investigated associations between emotional demands and psychological well-being [43, 44] and a study from Duarte and colleagues [44] distinguishes between emotional demands from clients and co-workers. Since this study focuses on emotional demands in veterinary practice, it may be speculated that the emotional demands experienced by employees in clinical veterinary practice are different from the types of emotional demands that are experienced by human service workers. However, according to a study by Dow et al. [12], the task of having to deal with bereaved pet owners constitutes a large aspect of the emotional demands in clinical veterinary practice, which implies that the emotional demands experienced by veterinary staff are interpersonal in their essence, and, hence, similar to the types of emotional demands experienced by e.g. human service workers.

The results of this study are important in terms of preventing negative health-related outcomes from adverse psychosocial working conditions, in particular, high emotional demands in combination with the four job demands that we have focused on in this study. This may be particularly relevant in clinical veterinary practice since we observe a higher prevalence of depression among employees in this sector (15.1%) than in the general population in Denmark (9.0%) [45]. To prevent the proliferation of mental health disorders among veterinary employees, it will, therefore, be pertinent for owners and managers in clinical veterinary practice to take preventive action if they become aware of combinations of high job demands in the psychosocial work environment.

These results are particularly relevant as a qualitative study among Canadian veterinarians reported that high stress and/or mental health problems were perceived to be associated with reduced quality of care [46]. Accordingly, these findings suggest that a stressful psychosocial work environment not only may have negative consequences on the mental health of employees in veterinary practice but also – indirectly via reduced mental health – on the quality of the delivered veterinary care services [46].

Against this background, it is relevant for work environment authorities and managers in clinical veterinary practice to encourage attention towards the psychosocial work environment. Not only to promote mental health among the employees but also to enhance the quality of the veterinary care services delivered in clinical practice. One potential avenue for promoting a healthy work environment may be to ensure the presence of job resources, such as leadership support, organizational justice, and social support in veterinary clinics. Previous findings show that the presence of job resources such as leadership support, organizational justice, and possibilities for development can buffer the risk of negative health-related outcomes that are associated with exposure to adverse working conditions [40, 47, 48]. Indeed, this approach aiming at boosting job resources to enhance the capacity of employees in the clinical veterinary sector to deal with high job demands may be promising. According to a study by Bakker and Sanz-Vergel [49], emotional demands may indeed turn into a so-called challenge demand [50], when employees have access to sufficient job resources, such as social support, autonomy, and feedback. A psychosocial work environment with adequate job resources may therefore not only prevent negative health-related outcomes from emotional demands, but also foster motivational outcomes, such as work engagement and job-satisfaction [18, 49, 51] in clinical veterinary practice.

To ensure that initiatives to boost job resources at the clinical level are effective, it is recommended that these initiatives are based on a participatory approach where managers and employees cooperate to identify the most appropriate courses of action to improve the psychosocial work environment [52]. This will enhance the fit between the identified initiatives and the organizational context [52] and strengthen employee motivation to support and implement the agreed initiatives to improve the work environment [53]. These are important prerequisites for successful efforts to improve the work environment.

### Limitations and strengths

It is an important limitation of the study that it is based on a cross-sectional survey design. This implies that we measured job demands and symptoms of depression at the same point in time, which precludes the possibilities for causal inference due to the lack of temporal separation of independent and dependent variables. Another related limitation pertains to the possibility of reverse causality, as depressive symptoms not only result from job demands but may also influence and shape how such job demands are perceived [54, 55]. Accordingly, this entails a risk that survey response is affected by reporting bias. While we acknowledge that the emotional state in the response situation may bias response, it is also relevant to acknowledge the large body of literature that demonstrates prospective associations between psychosocial working conditions and mental health outcomes [30] and this literature supports the findings of the present study.

It is also a limitation that our study sample is relatively small (i.e., 885 participants), which implies that the analysis may be underpowered. Indeed, compared to previous studies using that same methodology in very large study populations [23, 40], we found comparatively high RERI-coefficients in the four analyses in this study, but none were statistically significant. This may imply that the analyses may be underpowered and further supports the conclusion that combinations of job demands are associated with an elevated risk of depression. While this may be considered a weakness of the study, it must also be recognized that (a) the present study is based on a large study population – consisting of approximately 16% of the background population – when compared to other studies within the veterinary profession and (b) that the aim of the present study was to analyze associations between variables describing psychosocial working conditions and mental health outcomes, which makes representativeness a lesser problem.

Although we conclude that these limitations do not compromise the validity of the findings, they must be taken into account in the interpretation of the results.

## Conclusion

The results of this study show that 15.1% of the sample have indications of depressive symptoms. Further, the findings show that emotional demands combined with other types of job demands in the psychosocial work environment were associated with an increased risk of depression among employees in clinical veterinary practice in Denmark. For all four combinations of job demands, the results indicated additive but not synergistic interaction effects between the investigated job demands in their association with depression. The results from the study are of importance for the veterinary profession in preventing adverse health-related outcomes among employees in clinical veterinary practice. Against these results, it could be relevant for future research to investigate the potential buffering effects of job resources on associations between job demands and risk of depression in employees in clinical veterinary practice.

## Electronic supplementary material

Below is the link to the electronic supplementary material.

Supplementary Material 1

Supplementary Material 2

## Data Availability

All data used and analyzed during this study are available on reasonable request.

## Abbreviations

JD-R model: Job Demands Resources Model
COR theory: Conservations of Resources Theory
DVA: The Danish Veterinary Association
VNU: Veterinary Nurses Union
DPQ: Danish Psychosocial Work Environment Questionnaire
MDI: Major Depression Inventory
OR: Odds Ratio
95% CI: 95% Confidence Interval
RERI: Relative Excess Risk due to Interaction
EMO: Emotional demands
QD: Quantitative demands
RC: Role conflicts
WP: Work pace
TW: Threats at work
