# Supplementary material for: Combinations of job demands are associated with increased risk of depression in clinical veterinary practice: a cross-sectional study
Source: Ir Vet J. 2024 Dec 27;77:23. doi: 10.1186/s13620-024-00284-x (PMC11673828; doi:10.1186/s13620-024-00284-x)
Supplement: Supplementary file 1 — Supplementary Material 1 [file 13620_2024_284_MOESM1_ESM.docx]

**Appendix I**

**measurement of Study variables**

The internal consistency, or reliability, of question in the scales about *Emotional demands, Quantitative demands and Role conflicts* was measured with Cronbach’s alpha. Cronbach’s alpha results range from 0 to 1, where a Cronbach’s alpha of .70 and above is considered a satisfactory level of internal reliability. The scale about *Work pace* consists of two items and the reliability was therefore measured with Pearson’s r. Pearson’s r range from -1 to 1, where a Pearson’s r of 0.50 suggests a strong positive association.

**THE DANISH PSYCHOSOCIAL WORK ENVIRONMENT QUESTIONNAIRE (DPQ)**

Psychosocial working conditions were assessed using the The Danish Psychosocial Work Environment Questionnaire (DPQ) ([1](#_ENREF_1)):

*Emotional demands* was measured using a three-item scale. Cronbach’s alpha: 0.81.

1. Are you placed in emotionally demanding situations at work?
2. As a result of your work, do you come into contact with people who counteract your efforts or are aggressive towards you?
3. Do you have to deal with relationships that are emotionally challenging?

Response options: Always; Often; Sometimes; Rarely; Never/almost never

*Quantitative demands* was measured using a four-item scale. Cronbach’s alpha: 0.87.

1. How often is it the case that you do not have time to complete all your work tasks?
2. How often do you receive unscheduled work tasks that place you under time pressure?
3. How often do you have deadlines that are hard to meet?
4. Do you get behind with your work?

Response options: Always; Often; Sometimes; Rarely; Never/almost never

*Role conflicts* was measured using a four-item scale. Cronbach’s alpha: 0.79.

1. Do you have to do things in your work that you feel should be done differently?
2. Are there any conflicting demands in your work?
3. Does your job involve tasks that conflict with your personal values?
4. Do you sometimes have to end a task even though you do not feel you have completed it?

Response options: To a very large extent, To a large extent, Somewhat, To a low extent, To a very low extent

*Work pace* was measured using a two-item scale. Pearson’s r: 0.60.

1. Is the pace of work so fast that it affects the quality of your work?
2. Do you have to work very fast?

Response options: Always; Often; Sometimes; Rarely; Never/almost never

*Threats at work* was measured with the following item:

1. Have you been exposed to work-related threats during the last 12 months?
   *('Threats' denotes verbal or written threats or threatening behaviour)*

Response options: Yes, daily or almost daily; Yes, weekly;Yes, monthly; Yes, occasionally; No

**THE MAJOR DEPRESSION INVENTORY (MDI)**

Depressive symptoms were assessed using the Major Depression Inventory (MDI) ([2](#_ENREF_2)). The MDI consists of 12 items assessing the presence of depressive symptoms during the last two weeks ([2](#_ENREF_2)). Response options ranged from 0 (the symptom has not been present at all) to 5 (the symptom has been present all of the time). The MDI sum score ranges from 0–50 points, as for two pairs of items only the higher score is considered. The algorithm for calculating scores on the MDI is described elsewhere ([2](#_ENREF_2)). A clinical validation study has previously shown that an MDI-score ≥ 21 indicates a clinical depressive disorder ([2](#_ENREF_2)), and, accordingly, we used this MDI-score for defining the presence of depressive symptoms in this study. The following questions were used to assess depressive symptoms.

1. Have you felt low in spirits or sad?
2. Have you lost interest in your daily activities?
3. Have you felt lacking in energy and strength?
4. Have you felt less self-confident?
5. Have you had a bad conscience or feelings of guilt?
6. Have you felt that life wasn’t worth living?
7. Have you had difficulty in concentrating, e.g., reading the newspaper or watching television?

8a. Have you felt very restless?

8b. Have you felt subdued?

1. Have you had trouble sleeping at night?

10a. Have you suffered from reduced appetite?

10b. Have you suffered from increased appetite?

Response options: All the time; Most of the time; Slightly more than half the time; Slightly less than half the time; Some of the time; At no time

**VARIABLES ON OCCUPATIONAL AND DEMOGRAPHIC BACKGROUND CHARACTERISTICS**

Occupational and demographic background characteristics were measured in the study questionnaire. Demographic background characteristics were; gender and age. Occupational characteristics were; current job position (clinical owner, employed veterinarian, veterinary nurse, veterinary nurse student and other) and type of clinical practice (equine practice, small animal practice, production practice or mixed practice).

**References**

1. Clausen T MI, Christensen KB, Bjorner JB, Poulsen OM, Maltesen T, Borg V, Rugulies R. The Danish Psychosocial Work Environment Questionnaire (DPQ): Development, content, reliability and validity. Scand J Work Environ Health 2019;no. 4 356-69.

2. Bech P, Timmerby N, Martiny K, Lunde M, Soendergaard S. Psychometric evaluation of the Major Depression Inventory (MDI) as depression severity scale using the LEAD (Longitudinal Expert Assessment of All Data) as index of validity. BMC Psychiatry. 2015;15:190.
