# Supplementary material for: Combinations of job demands are associated with increased risk of depression in clinical veterinary practice: a cross-sectional study
Source: Ir Vet J. 2024 Dec 27;77:23. doi: 10.1186/s13620-024-00284-x (PMC11673828; doi:10.1186/s13620-024-00284-x)
Supplement: Supplementary file 2 — Supplementary Material 2 [file 13620_2024_284_MOESM2_ESM.docx]

**Appendix 2**

**Appendix Table 1:** **Risk of depression stratified by sociodemographic groups. Results from logistic regression analysis**

|  | **At risk N (%)** | **Cases N (%)** | **OR** | **95 % CI** |
| --- | --- | --- | --- | --- |
| **Gender** |  |  |  |  |
| Male | 74 (9.4) | 5 (0.6) | 1 | Ref. |
| Female | 711 (90.6) | 110 (14.0) | 2.5 | 1.0 to 6.4 |
| **Age** |  |  |  |  |
| < 25 years | 44 (5.6) | 6 (0.8) | 1 | Ref. |
| 25-34 years | 303 (38.4) | 64 (8.1) | 1.7 | 0.69 to 4.19 |
| 35-44 years | 220 (27.9) | 36 (4.6) | 1.2 | 0.49 to 3.15 |
| 45-54 years | 146 (18.5) | 12 (1.5) | 0.6 | 0.20 to 1.61 |
| > 55 years | 76 (9.6) | <5 | 0.1 | 0.01 to 0.73 |
| **Job position** |  |  |  |  |
| Clinic owner | 74 (9.4) | <5 | 1 | Ref. |
| Employed veterinarian | 344 (43.6) | 50 (6.3) | 3.0 | 1.0 to 8.5 |
| Veterinary nurse | 237 (30.0) | 41 (5.2) | 3.7 | 1.3 to 10.6 |
| Veterinary nurse student | 71 (9.0) | 14 (1.8) | 4.3 | 1.3 to 13.8 |
| Other | 63 (8.0) | 10 (1.3) | 3.3 | 1.0 to 11.1 |
| **Type of clinical pratice** |  |  |  |  |
| Mixed practice | 78 (9.9) | 10 (1.3) | 1 | Ref. |
| Small animal practice | 636 (80.9) | 102 (13.0) | 1.3 | 0.6 to 2.6 |
| Production practice | 37 (4.7) | 5 (0.6) | 1.1 | 0.3 to 3.4 |
| Equine practice | 35 (4.5) | <5 | 0.2 | 0.0 to 1.6 |
